# Thoracic gas compression during forced expiration is greater in men than women

**DOI:** 10.14814/phy2.14404

**Published:** 2020-03-24

**Authors:** Elizabeth A. Gideon, Troy J. Cross, Brooke E. Cayo, Aaron W. Betts, Dallin S. Merrell, Catherine L. Coriell, Lauren E. Hays, Joseph W. Duke

**Affiliations:** ^1^ Department of Biological Sciences Northern Arizona University Flagstaff AZ USA; ^2^ Department of Cardiovascular Diseases Mayo Clinic Rochester MN USA; ^3^ Griffith University Menzies Health Institute Queensland Brisbane QLD Australia

**Keywords:** alveolar pressure, area under the curve, flow volume loop, lung recoil pressure, respiratory mechanics

## Abstract

Intrapleural pressure during a forced vital capacity (VC) maneuver is often in excess of that required to generate maximal expiratory airflow. This excess pressure compresses alveolar gas (i.e., thoracic gas compression [TGC]), resulting in underestimated forced expiratory flows (FEFs) at a given lung volume. It is unknown if TGC is influenced by sex; however, because men have larger lungs and stronger respiratory muscles, we hypothesized that men would have greater TGC. We examined TGC across the “effort‐dependent” region of VC in healthy young men (*n* = 11) and women (*n* = 12). Subjects performed VC maneuvers at varying efforts while airflow, volume, and esophageal pressure (P_OES_) were measured. Quasistatic expiratory deflation curves were used to obtain lung recoil (P_LUNG_) and alveolar pressures (i.e., P_ALV_ = P_OES_–P_LUNG_). The raw maximal expiratory flow–volume (MEFV_raw_) curve was obtained from the “maximum effort” VC maneuver. The TGC‐corrected curve was obtained by constructing a “maximal perimeter” curve from all VC efforts (MEFV_corr_). TGC was examined via differences between curves in FEFs (∆FEF), area under the expiratory curves (∆A_EX_), and estimated compressed gas volume (∆VGC) across the VC range. Men displayed greater total ∆A_EX_ (5.4 ± 2.0 vs. 2.0 ± 1.5 L^2^·s^−1^; *p* < .001). ∆FEF was greater in men at 25% of exhaled volume only (*p* < .05), whereas ∆VGC was systematically greater in men across the entire VC (main effect; *p* < .05). P_ALV_ was also greater in men throughout forced expiration (*p* < .01). Taken together, these findings demonstrate that men display more TGC, occurring early in forced expiration, likely due to greater expiratory pressures throughout the forced VC maneuver.

## Graphical abstract

We sought to examine whether sex influences the degree to which thoracic gas compression (TGC) underestimates forced expiratory flows at a given lung volume during forced expiration. Men displayed greater TGC than women, particularly at 25% of the forced expired volume. This greater TGC was likely due to greater expiratory pressures generated by men throughout the maneuver. Failure to appropriately account for TGC results in a greater underestimation of expiratory airflow in men, primarily during the early phase of forced expiration.

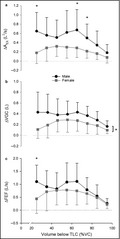

## 1 INTRODUCTION

The forced vital capacity (VC) maneuver provides substantive information about an individual's respiratory health and function (Miller et al., 2005). From this maneuver, the maximal expiratory flow–volume (MEFV) curve is obtained. The envelope inscribed by the MEFV curve represents the upper boundary of an individual's range of maximally achievable expiratory flows which, if greatly reduced, may indicate underlying pathophysiology (Miller et al., 2005). To obtain the MEFV curve, an individual must produce large, positive swings in intrapleural pressure (often approximated by esophageal pressure [P_OES_] (Gillespie, Lai, & Hyatt, 1973; Hurewitz, Sidhu, Bergofsky, & Chanana, 1984; Hamid, Shannon, & Martin, 2005)). These pressures, however, are often far in excess of those required to generate maximum expiratory airflow (i.e., expiratory flow limitation) (Ingram & Schilder, 1966; Sharafkhaneh et al., 2007). Under these circumstances, alveolar gas is compressed in a manner proportional to the excess pressure developed during the forced expiration. The precipitating effect of such thoracic gas compression (TGC) is that forced expiratory flows (FEFs) measured at the mouth may be greatly underestimated at a given lung volume (Cross et al., 2018; Guenette et al., 2010; Ingram & Schilder, 1966; Sharafkhaneh et al., 2007; Sharafkhaneh, Officer, Goodnight‐White, Rodarte, & Boriek, 2004). Indeed, others have clearly demonstrated that TGC artifact results in an underestimation of bronchodilator efficacy (Sharafkhaneh et al., 2007) and an overestimation of ventilatory constraint during exercise (Guenette et al., 2010). It follows from the above that adjusting the MEFV curve for TGC artifact has important implications for correct interpretation of pulmonary function (Cross et al., 2018). Interestingly, these previous studies provided comments on intra‐subject variability of TGC within their experimental groups; however, no study to date has deliberately examined how participant sex may affect the degree of TGC observed during forced VC maneuvers (Cross et al., 2018; Guenette et al., 2010; Sharafkhaneh et al., 2007).

There are three principal factors which determine the magnitude of TGC during forced expiration: (a) the density of the respired gas (Cross et al., 2018); (b) the magnitude of alveolar pressure (P_ALV_); and (c) the absolute volume of gas within the lung at the time of compression (Ingram & Schilder, 1966; Jaeger & Otis, 1964). Notwithstanding abrupt changes in barometric pressure (e.g., sojourning at high altitude) and/or the density of inhaled gas (i.e., room‐air vs. helium‐oxygen), it is primarily the latter two factors which determine TGC in the acute laboratory setting. With regard to the potential effect of sex, it is well‐established that, on average, men have stronger respiratory muscles (Chen & Kuo, 1989). Thus, men have a greater capacity to generate greater alveolar pressures during forced expiration than women. Additionally, men typically present with greater static (absolute) lung volumes than women. It follows from the above that men have a greater possibility of incurring TGC during forced VC maneuvers than their female counterparts. Given the growing abundance of research examining differences in respiratory physiology between men and women (Sheel & Guenette, 2008; Dominelli et al., 2013; Dominelli, Molgat‐Seon, et al., 2015; Dominelli, Render, et al., 2015), it would be prudent to determine whether sex differences exist in the degree of TGC observed during forced expirations.

Therefore, the purpose of this study was to compare the magnitude of TGC across the VC range between men and women with normal pulmonary function. Seeing that the difference in absolute lung volume between sexes is largest at high lung volumes (i.e., nearing total lung capacity), and that the pressure generating capacity of expiratory muscles is greatest at full inflation (Agostoni & Hyatt, 1986), we hypothesized that men would display larger TGC artifact than women primarily at higher fractions of VC during forced expiration.

## 2 METHODS

### 2.1 Ethical approval, screening, and pulmonary function

Fifty‐nine subjects, aged 18–35 years, completed the informed consent approved by the Northern Arizona University Institutional Review Board. Upon completion of the informed consent, all 59 subjects completed the screening and pulmonary function testing. Sixteen participants did not meet the inclusion criteria for normal pulmonary function (i.e., >80% predicted on forced VC, forced expiratory volume in 1 s, the ratio of forced expiratory volume in 1 s to forced VC, and forced expiratory airflow from 25% to 75% of VC). If a subject had only a single value less than 80% of predicted, they were allowed to participate if the magnitude of that parameter exceeded the lower limit of normal (Quanjer et al., 2012). Three participants withdrew from the study after visit 1, one participant did not finish due to illness, and data from 16 participants were not included due to poor quality of graded, forced VC efforts (i.e., too few maneuvers, insufficient data through range of efforts, etc.). Altogether, 23 participants (11 men; 12 women) completed all aspects of the study. Only data from these participants were used for further analysis. All participants had no known history of metabolic, pulmonary, or cardiovascular disease.

### 2.2 Experimental design

Participants visited the laboratory on two occasions separated by at least 24 hr and no more than 10 days. During the first visit, the details of the study were discussed and participants provided their written and informed consent. Routine spirometry was then performed, including slow and forced vital capacity maneuvers (CPFS/D Spirometer; MedGraphics). Pulmonary function data were obtained and reported according to the American Thoracic Society/European Respiratory Society guidelines (Miller et al., 2005). On the second visit, participants were instrumented with an esophageal balloon, and allowed to rest quietly for ~10 min before experimental data were collected. Participants then completed 10–15 graded expiratory vital capacity (GVC) maneuvers ranging from 100% to 5% of maximal expiratory efforts. These efforts were used to construct a “maximal perimeter” MEFV curve and thereby adjust for TGC artifact during forced expiration (see section *Graded Vital Capacity Maneuvers* for more details). Participants also completed a series (2–3) of quasistatic expiratory deflation curves, from which static recoil pressure of the lungs (P_LUNG_) was estimated across the entire VC range.

### 2.3 Respiratory pressures, flows, and volumes, and data acquisition

#### 2.3.1 Respiratory pressures

Upon arrival to the laboratory for Visit 2, participants were instrumented with a balloon‐tipped esophageal catheter (47‐9005; Ackrad Laboratory). Following application of a topical anesthetic (2% lidocaine HCl) to numb the naris and nasopharynx, the balloon catheter was passed through the nares and all the way into the stomach (i.e., ~60–65 cm distal to the tip of the nostril). Next, the residual air was evacuated from the balloon via a Valsalva maneuver or a forceful cough with an open glottis, after which the balloon was inflated with ~1 to 1.5 ml of air. After confirming the balloon was positioned in the stomach (i.e., diaphragmatic “bumps” during inspiration), the balloon catheter was withdrawn until a persistent negative deflection was observed during inspiratory effort. Final adjustments of balloon position was guided by the “occlusion” test, wherein balloon depth was modified such that ∆P_OES_/∆ mouth pressure (P_M_) was within 0.98–1.02 (Baydur, Behrakis, Zin, Jaeger, & Milic‐Emili, 1982). P_M_ and P_OES_ were measured by two separate differential pressure transducers with a range of either ± 140 cmH_2_O (*n* = 7; series 1110 Pneumotach amplifier; Hans Rudolph, Shawnee, KS, USA) or ± 352 cmH_2_O (*n* = 16; HSCSNBN005PDAA5, Honeywell International Inc., NJ, USA). The pressure transducers were calibrated using a water manometer before each visit.

#### 2.3.2 Respiratory flow and volume

For the duration of Visit 2, participants remained seated, upright resting posture while breathing on a mouthpiece attached to a two‐way non‐rebreathing valve (2700 series; Hans Rudolph). P_M_ and respiratory gases were sampled via a lateral port on the mouthpiece. Respiratory gases were analyzed via mass spectrometry (Marquette MGA 1100, MA Tech Services). Inspired and expired airflow were measured separately via two linear pneumotachographs (4813/3813 series; Hans Rudolph) and respective volumes were computed via numerical integration of the flows recorded by each device. The pneumotachograph on the expiratory side of the respiratory circuit was heated to 37°C. Data were sampled at 100 Hz and acquired throughout the entire visit using the Beck Integrative Physiological System (BIPS; KCBeck Physiological Consulting).

### 2.4 Graded vital capacity maneuvers

While seated at rest, participants completed a minimum of 10–15 GVC maneuvers at expiratory efforts ranging from 5% to 100% of an individual's perceived “maximum effort.” In brief, participants were instructed to rapidly inhale to total lung capacity (TLC), followed immediately (without pause) by a forced expiration to residual volume (RV). Participants completed this initial expiratory maneuver at 100% of their maximal voluntary effort. Immediately following this effort, participants rapidly inhaled to TLC and, without pause, performed a full exhalation to RV at a slightly lesser expiratory effort. The participants were instructed to decrease effort by roughly 10% for each subsequent maneuver. Participants performed 3–4 maneuvers consecutively and then were given a break before performing additional sets of maneuvers. Great care was taken to ensure that adequate GVC maneuvers were obtained across the desired range of expiratory efforts, each beginning at TLC and terminating at RV. During each maneuver, the investigators visually inspected the flow–volume and pressure data in real time. In the event that a poor effort was given, participants were asked to repeat the GVC at the graded effort in question. The *raw*, uncorrected MEFV curve was obtained from the single effort with the largest VC, and most positive peak expiratory P_OES_, from the GVC maneuvers performed at ~100% of maximum effort; this raw curve is referred to hereafter as MEFV_raw_. The TGC‐*corrected* MEFV envelope was obtained by taking the highest FEF observed at a given lung volume across all GVC efforts (Cross et al., 2018; Cross, Winters, Sheel, & Sabapathy, 2014; Guenette et al., 2010; Olafsson & Hyatt, 1969). This “maximal perimeter” MEFV curve is thought to mitigate the issue(s) associated with TGC during forced expirations (Cross et al., 2014, 2018; Guenette et al., 2010; Olafsson & Hyatt, 1969). As such, we hereafter refer to the “maximal perimeter” flow–volume envelope as the TGC‐*corrected* MEFV curve (MEFV_corr_).

### 2.5 Quasistatic expiratory deflation curves of the lungs

**Figure 1:**
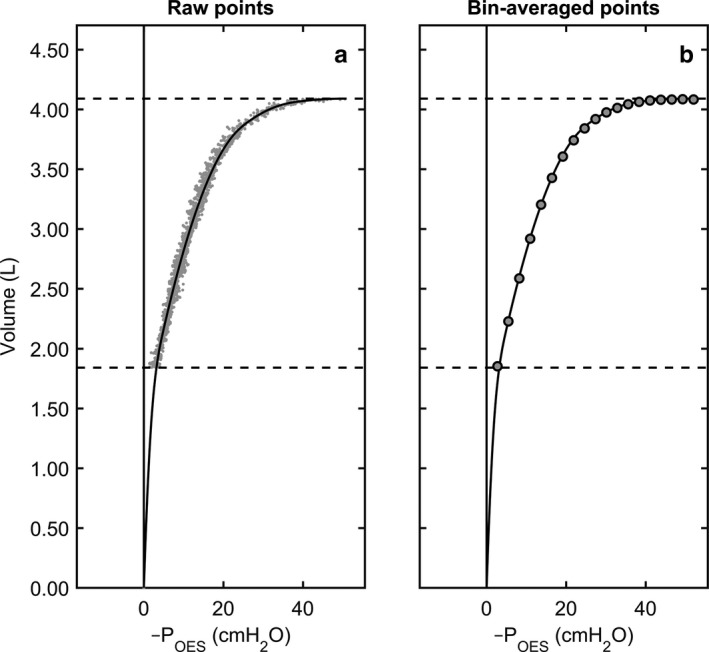
Illustration of methodology used to determine recoil pressure of the lung (P_LUNG_). (a) Raw P_OES_ versus volume data when flow was very low (<100 ml·s^−1^). (b) Raw data were then bin‐averaged into 20 equidistant “pressure” bins along the *x*‐axis and a shape‐constrained, piecewise cubic spline was fit to the bin‐averaged data allowing for a continuous approximation of the P_LUNG_–volume relationship to be obtained

While the participants were completing the 10–15 GVC efforts as described above, particular attention was paid to the VC maneuvers performed at exceedingly low expiratory flows (<150 ml·s^−1^). Pressure, flow, and volume data from these very low flow deflations were taken as surrogates for quasistatic expiratory deflation curves of the lung, similar to that done by others (Lu et al., 1999; Servillo et al., 1997). The static‐recoil pressure of the lung (P_LUNG_) was estimated as the negative sign of P_OES_ during the points of each GVC where expiratory flow was <150 ml·s^−1^. That is, if P_LUNG_ = P_ALV_ – P_PL_, and if P_OES_ is a valid surrogate for P_PL_ (Gillespie et al., 1973; Hamid et al., 2005; Hurewitz et al., 1984), then P_LUNG_ = –P_OES_ whenever P_ALV_ is zero. Importantly, relaxation of the respiratory muscles is not required to estimate P_LUNG_ during these very slow deflations. For example, while P_PL_ may be determined by P_ALV_–P_LUNG_, it is also true that P_PL_ is contributed by the subtraction of net respiratory muscle pressure (P_MUS_) from static recoil pressures of the chest wall (P_CW_), such that: P_PL_ = P_CW_–P_MUS_, where a *positive* P_MUS_ denotes *inspiratory* effort. In turn, when P_ALV_ is 0 cmH_2_O and the glottis is held open, the continuity of pressures acting across the chest wall and lungs can be written as: P_CW_–P_MUS_ = P_PL_ = –P_LUNG_. This equation illustrates that persistent inspiratory “braking” during *very* slow deflations does not interfere with approximating P_LUNG_ from –P_PL_ (or –P_OES_) so long as P_ALV_ is sufficiently close to atmospheric (0 cmH_2_O). Certainly, even with a conservatively high estimate of expiratory airways resistance of 5 cmH_2_O∙L^‐1^∙s, the prevailing P_ALV_ would be at most 0.75 cmH_2_O above atmospheric during GVC efforts where instantaneous flows were less than 150 ml·s^−1^. Thus, we assumed that P_ALV_ approximated 0 cmH_2_O during the *very* slow deflations. In turn, the –P_OES_ (i.e., P_LUNG_) data was plotted as a function of lung volume for each subject (Figure 1a). Given the known issues with obtaining reliable estimates of lung recoil pressure at volumes below FRC in healthy individuals (Mead, Turner, Macklem, & Little, 1967; Milic‐Emili, Mead, Turner, & Glauser, 1964), we limited our analysis to consider only P_LUNG_‐volume data pairs between 100% of VC (i.e., TLC) and 40% of VC. Data reduction of raw P_LUNG_ data was performed by bin‐averaging values into 20 equidistant “pressure” bins along the *x*‐axis (Figure 1b). A shape‐constrained, piecewise cubic spline was fit to the bin‐averaged data, and a continuous approximation of the P_LUNG_–volume relationship was obtained. The number of knots chosen for this spline varied among subjects, ranging between 4 and 10 knots (median of 6). The choice of the number of knots was largely guided by visual inspection of the resulting fit and of the root‐mean‐square error of residual scores. The spline was “shape‐constrained” to be a monotonically increasing, downwardly concaved function with a smooth third‐order derivative. A further constraint was imposed on the spline, such that it was forced to run through the origin. This latter constraint had the effect of gracefully extrapolating data from 40% down to 0% VC.

### 2.6 Thoracic gas compression

**Figure 2:**
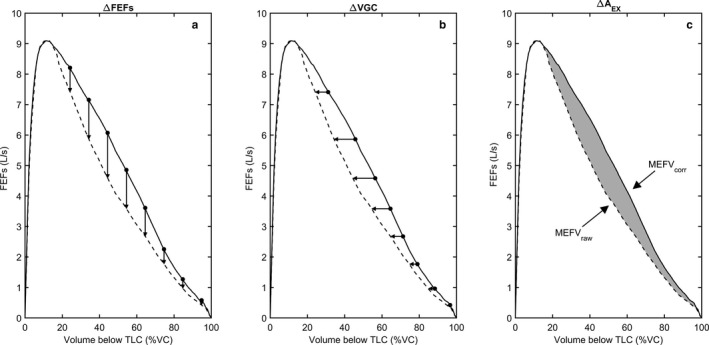
Illustration of methodology used to study thoracic gas compression (TGC) in three ways. (a) TGC was calculated as the difference in instantaneous forced expiratory flow (∆FEF) between MEFV_raw_ and MEFV_corr_ curves. (b) TGC was also defined as the horizontal difference between similar FEFs of the MEFV_raw_ and MEFV_corr_ curves, which provided an index of the volume of gas compressed (∆VGC). (c) TGC was also computed as the integrated area between the MEFV_raw_ and MEFV_corr_ curves (∆A_EX_)

Because we did not make measurements inside of a whole‐body plethysmograph, we can only be confident about our measures of TGC beyond the lung volume at which peak expiratory airflow occurred. Accordingly, we quantified TGC artifact in three different ways from 20% to 100% of VC. First, TGC was calculated as the difference in instantaneous forced expiratory flows (∆FEF) between MEFV_raw_ and MEFV_corr_ curves at a given lung volume (Figure 2a). In this manner, the greater that FEF was underestimated by TGC artifact, the larger was the absolute value of ∆FEF. Second, TGC was defined as the horizontal distance between similar FEFs of the MEFV_raw_ and MEFV_corr_ curves (Figure 2b) (Sen, Bartu, Yildiz, & Kose, 2009; Walamies, 1998). This measure of TGC provided an index of the volume of gas compressed at a given percent of VC during forced expiration (∆VGC). Lastly, the final measure of TGC was computed as the integrated area between MEFV_raw_ and MEFV_corr_ curves (∆A_EX;_ Figure 2c). The relative (%) magnitude of ∆FEF was obtained by expressing ∆FEF as a fraction of the FEF on the MEFV_corr_ curve at the corresponding lung volume.

### 2.7 “Compression‐free” pressure–volume data

Having thus computed the ∆VGC at each lung volume, it was possible to *correct* pressure–volume data obtained during the 100% GVC effort; that is, the VC effort with the largest TGC artifact. This correction was achieved by point‐by‐point subtraction of ∆VGC from the expired volume during the corresponding VC maneuver. In so doing, we were able to attribute the discrete values for each pressure waveform (P_M_, P_OES_, P_ALV_, etc.) with the correct lung volume, had no gas compression occurred during the forced expiratory effort. This volume correction was important given that we intended to compare the magnitudes of expiratory effort during forced expirations at similar lung volumes between sexes.

### 2.8 Data analysis

The initial data analysis step included correction of the dynamic response of commercial esophageal balloon catheters using exponential model correction, as has been previously described (Cross, Beck, & Johnson, 2016). Flow and pressure data obtained during each GVC effort were anchored to total lung capacity and bin‐averaged into 1,000 equidistant volume bins, whose range spanned each participant's largest VC. Any effort that differed from the largest VC by > 10% was excluded from subsequent analyses. Bin‐averaging data in this manner simplified the process of constructing the MEFV_corr_ curve, and provided homogenous datasets across participants. Importantly, given the nature by which the “maximal perimeter” MEFV curve is constructed, it is entirely possible that between two consecutive flow–volume data pairs, the magnitude of TGC artifact (e.g., ∆FEF) may vary spuriously from zero to some nontrivial value. In which case, one may erroneously conclude that no gas compression was evident if only the former lung volume were considered, and not the latter. Thus, once secondary variables were calculated (i.e., P_LUNG_, P_ALV_, ∆FEF, ∆VGC, etc.), flow and pressure data were further bin‐averaged in into smaller, equidistant volume increments representing a width of 10% of VC, that is, 20%–30% of VC, and displayed as the central value of the bin, which would be 25% of VC in the aforementioned example. This bin width was chosen because it was large enough to provide robust estimates of TGC artifact (see above), while also being narrow enough to facilitate meaningful comparisons across lung volumes between sexes.

### 2.9 Statistical analysis

Participant characteristics, baseline pulmonary function data, and total A_EX_ were compared between sexes using independent samples *t* tests. To examine possible interactions between sex and lung volume on our three measures of TGC artifact (i.e., ∆A_EX_, ∆VGC, and ∆FEF), a repeated measures analyses of variance was performed using the MIXED function in SPSS (Version 25, IBM Corporation). A similar approach was used to examine possible interactions between sex and lung volume for P_OES_, P_LUNG_, and P_ALV_. Pairwise comparisons were adjusted for multiple comparisons using the Tukey–Sidak post hoc adjustment. All statistical analyses were considered significant if *p* < .05.

## 3 RESULTS

### 3.1 Anthropometrics and spirometry

Anthropometrics and spirometry data, collected to determine inclusion in the study, for all participants are presented in Table 1. In our group of participants, men were taller and heavier than the women (*p* < .05). The spirometry data presented here demonstrate that our participants had normal lung function based on values being >80% of predicted. Likewise, differences between men and women were as expected, whereby men displayed greater lung volumes and larger forced expiratory flows (i.e., forced vital capacity, slow vital capacity, and forced expired volume in 1 s, etc.; *p* < .05). When expressed as a percent of age‐predicted values, pulmonary function was comparable between men and women except for forced expiratory flow at 75% of vital capacity was greater in women compared to men (*p* < .05).

**Table 1:** Anthropometrics and pulmonary function data obtained during initial screening

	Male (*n* = 11)	Female (*n* = 12)
Age, yr	23.1 ± 4.8	21.2 ± 1.0
Height, cm	181.5 ± 4.9	165.0 ± 5.5*
Mass, kg	84.9 ± 11.0	60.5 ± 6.1*
BMI, kg·m^−2^	25.7 ± 2.8	22.3 ± 2.9*
Spirometry		
FVC, liters	6.0 ± 0.5 (105 ± 6)	4.0 ± 0.4* (101 ± 11)
FEV_1_, liters	4.9 ± 0.5 (103 ± 6)	3.6 ± 0.2* (106 ± 8)
FEV_1_/FVC	82 ± 8 (97 ± 9)	91 ± 7* (104 ± 7)*
PEF, L·s^−1^	10.8 ± 1.3 (102 ± 13)	8.1 ± 1.1* (115 ± 17)
FEF_25_, L·s^−1^	9.2 ± 1.8 (107 ± 23)	7.1 ± 0.8* (118 ± 16)
FEF_50_, L·s^−1^	6.0 ± 1.9 (103 ± 32)	5.1 ± 1.0 (106 ± 21)
FEF_75_, L·s^−1^	2.4 ± 1.0 (98 ± 27)	2.6 ± 0.7 (130 ± 37)*
FEF_25−75_, L·s^−1^	5.1 ± 1.5 (99 ± 26)	4.5 ± 0.9 (113 ± 23)
SVC, liters	6.1 ± 0.6 (107 ± 8)	3.9 ± 0.4* (100 ± 13)
IC, liters	4.0 ± 0.6 (107 ± 16)	2.6 ± 0.4* (100 ± 19)
ERV, liters	2.1 ± 0.5 (110 ± 23)	1.3 ± 0.4* (104 ± 31)

Note

All values are expressed and mean ± standard deviation (*SD*) and values in parentheses are mean ± *SD*% of predicted normal values.

Abbreviations: BMI, body mass index; ERV, expiratory reserve volume; FEF_25_, forced expiratory flow at 25% of FVC; FEF_25–75_, average forced expiratory flow from 25% to 75% of FVC; FEF_50_, forced expiratory flow at 50% of FVC; FEF_75_, forced expiratory flow at 75% of FVC; FEV_1_, forced expiratory volume in 1 s; FVC, forced vital capacity; IC, inspiratory capacity; PEF, peak expiratory flow; SVC, slow vital capacity.

* Denotes statistically significant difference from males, *p* < .05.

### 3.2 Magnitude and location of TGC

**Figure 3:**
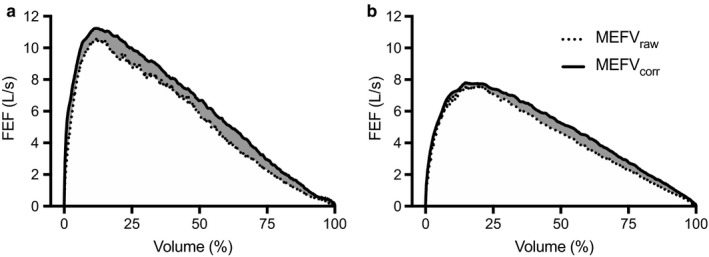
Average MEFV_raw_ (dotted line) and MEFV_corr_ (solid line) curves for men (a) and women (b). The grey shaded region represents the magnitude of total A_EX_

**Figure 4:**
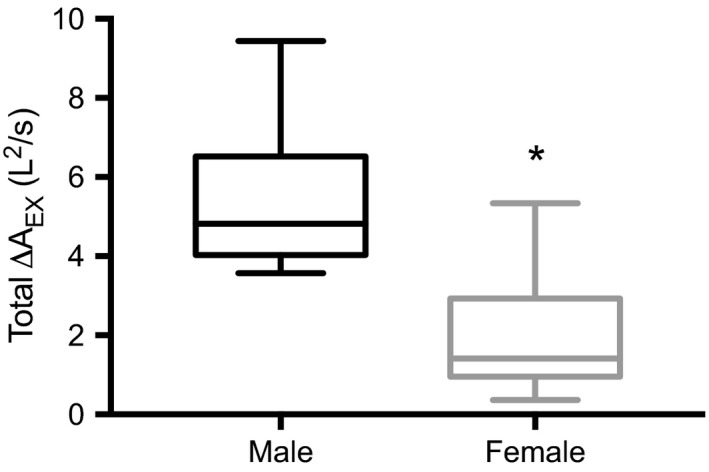
Boxplots of the total ∆A_EX_ in men and women. *Denotes a significantly greater change in area in men compared to women (*p* < .05)

**Figure 5:**
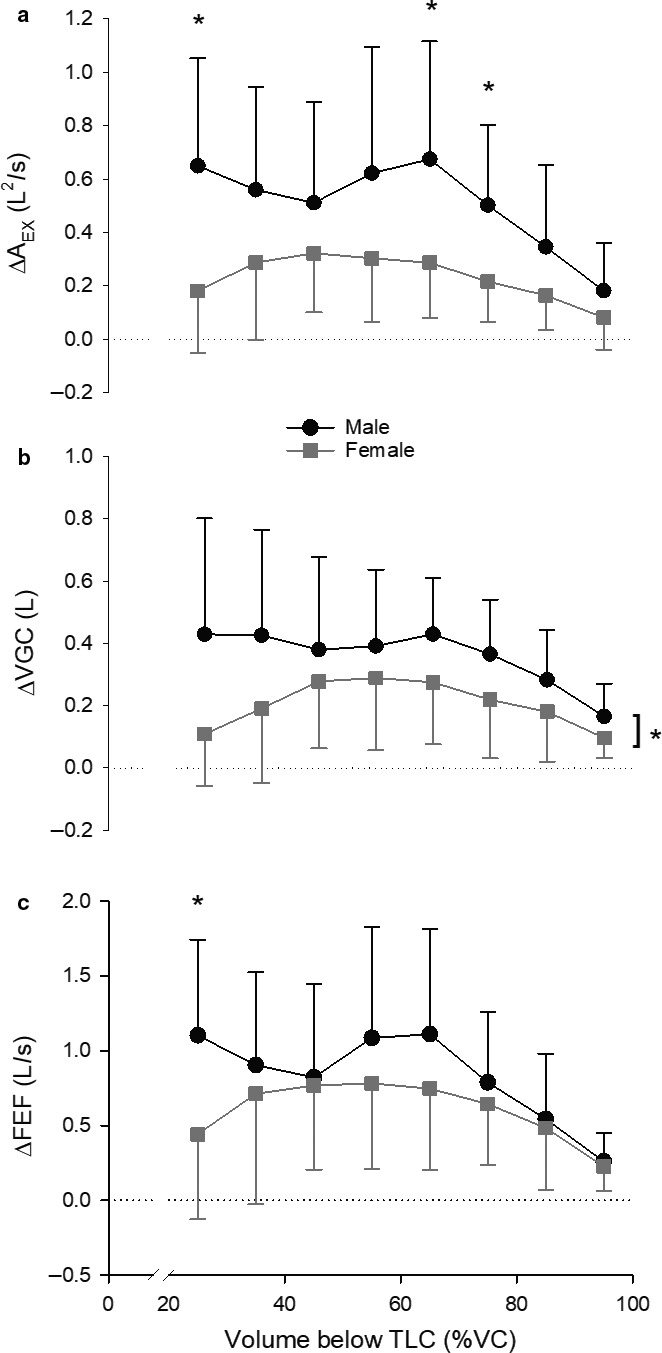
Mean ± *SD* ∆A_EX_ (a), ∆VGC (b), and ∆FEF (c) data for men (black circles) and women (grey squares) at 10% of VC increments. *Denotes a statistically significant difference between men and women at a given lung volume. Bracket in b denotes a significant main effect for sex

Figure 3 displays the average MEFV_raw_ and MEFV_corr_ curves for men and women (a and b, respectively). The sum total A_EX_ increased from MEFV_raw_ to MEFV_corr_ curves in men (32.6 ± 8.6 vs. 38.0 ± 9.3 L^2^/sec; *p* < .001) and women (16.3 ± 3.6 vs. 18.3 ± 4.1 L^2^/sec; *p* < .001). Total A_EX_ was greater in men than in women for both MEFV_raw_ and MEFV_corr_ (*p* < .001). ∆A_EX_ was greater in men than in women (Figure 4; *p* < .001). Figure 5 displays the three measures of TGC during forced expiration. Men had a greater ∆A_EX_ (Figure 5a) at 25%, 65%, and 75% of VC compared with women (*p* < .01). There was a main effect of sex for ∆VGC (Figure 5b), whereby ∆VGC was systematically greater than women throughout the VC range (*p* = .04). ∆FEF (Figure 5c) was significantly greater in men than in women at 25% of VC only (*p* = .01). Table 2 displays spirometry both with and without TGC correction. When expressed as absolute values, forced VC, forced expired volume in 1 s (FEV_1_), peak expiratory flow rate, and mid‐expiratory flows were greater in men than in women, regardless of the curve examined. Likewise, men displayed a significant improvement with TGC correction in all parameters other than forced VC, while women had a significant improvement in FEV_1_ and FEF at 50% and 75% of VC, and the average mid‐expiratory flow from 25%–75% of VC. Examination of the change in spirometry parameters revealed that only the change in FEF at 25% of VC was greater in men than in women when expressed in absolute units (*p* = .003) and as a percent of MEFV_corr_ (*p* = .01).

**Table 2:** Spirometry without and with TGC correction in male and female groups obtained during main study visit

	Male (*n = 11*)				Female (*n = 12*)			
	MEFV_raw_	MEFV_corr_	Abs change	% change	MEFV_raw_	MEFV_corr_	Abs change	% change
FVC, L	6.1 ± 0.6	6.2 ± 0.6	0.1 ± 0.1	1 ± 2	3.9 ± 0.3*	3.9 ± 0.3*	0.1 ± 0.1	1 ± 3
FEV_1_, L	4.7 ± 0.6	5.2 ± 0.6^†^	0.5 ± 0.2	10 ± 4	3.2 ± 0.4*	3.5 ± 0.4*†	0.3 ± 0.3	9 ± 8
FEV_1_/FVC, %	76 ± 8	84 ± 7^†^	8 ± 3	10 ± 4	83 ± 11	91 ± 9	8 ± 8	9 ± 8
PEFR, l·s−1	11.5 ± 2.3	12.0 ± 2.2^†^	0.6 ± 0.6	5 ± 5	8.0 ± 1.8*	8.2 ± 1.8*	0.2 ± 0.4	3 ± 4
FEF_25_, l·s^−1^	8.7 ± 2.1	10.0 ± 1.9^†^	1.3 ± 0.8	13 ± 8	7.0 ± 1.5*	7.4 ± 1.6*	0.4 ± 0.5*	5 ± 6*
FEF_50_, l·s^−1^	5.7 ± 1.8	6.7 ± 1.7^†^	0.9 ± 0.8	14 ± 12	4.5 ± 1.1	5.3 ± 1.3*†	0.7 ± 0.6	13 ± 11
FEF_75_, l·s^−1^	2.2 ± 0.8	3.0 ± 1.0^†^	0.8 ± 0.4	26 ± 9	2.2 ± 0.9	2.8 ± 1.0^†^	0.6 ± 0.4	22 ± 15
FEF_25−75_, l·s^−1^	5.7 ± 1.6	6.6 ± 1.6^†^	1.0 ± 0.5	15 ± 7	4.5 ± 1.1	5.3 ± 1.2*†	0.7 ± 0.5	13 ± 9

Note

All values are expressed and mean ± standard deviation (*SD*). Values in the MEFV_raw_ and MEFV_corr_ columns are the absolute values. Values in Abs change column is the change, that is, MEFV_corr_ ‐ MEFV_raw_, in absolute units while values in the % change column is the change expressed as a percent of MEFV_corr_.

Abbreviations: FEF_25_, forced expiratory flow at 25% of FVC; FEF_25–75_; FEF_50_, forced expiratory flow at 50% of FVC; FEF_75_, forced expiratory flow at 75% of FVC; FEV_1_, forced expired volume in 1 s; FEV_1_/FVC, ratio of FEV_1_ to forced vital capacity expressed as a percentage; FVC, forced vital capacity; PEFR, peak expiratory flow rate.

* Denotes a statistically significant difference between sexes, *p* < .05.

^†^ Denotes a statistically significant difference between MEFV_raw_ and MEFV_corr_, *p* < .05.

### 3.3 P_LUNG_, P_OES_, and P_ALV_

**Figure 6:**
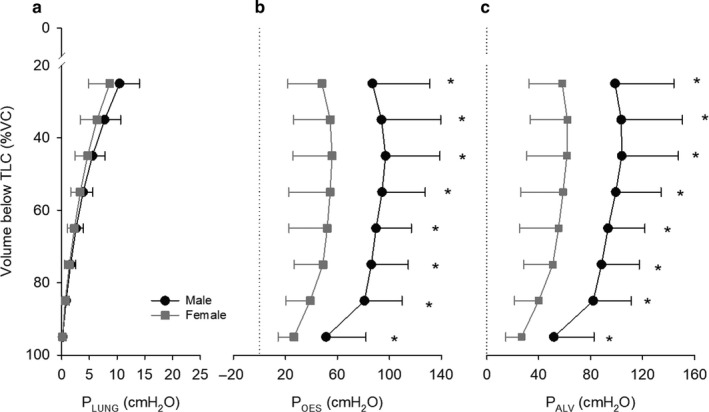
Average ± *SD* P_LUNG_ (a), P_OES_ (b), and P_ALV_ (c) for men (black circles) and women (grey squares) at 10% of VC increments. There was neither a significant interaction nor a significant effect of sex for P_LUNG_. *Denotes a statistically significant difference between men and women at a given lung volume

Mean P_LUNG_–volume curves for men and women are displayed in Figure 6a. There was no effect of sex on P_LUNG_ across the VC range. Figure 6 also displays P_OES_ (b) and P_ALV_ (c) for men and women at 10% of VC increments. Men had a significantly greater P_OES_ throughout the VC range that was analyzed (*p* < .05). Likewise, men had a significantly greater P_ALV_ than women at all lung volumes considered (*p* < .05).

## 4 DISCUSSION

The main findings of this study were that men displayed a greater magnitude of TGC artifact during forced expiration than women. Specifically, this larger magnitude of TGC artifact was primarily confined to the early portion (25%) of the forced expiratory volume (Figure 5a). For the remaining portion of forced expiration, the magnitude of TGC determined using ∆A_EX_ was, on average, greater in men than in women, but only statistically significant at 65% and 75% of VC. TGC quantified via ∆FEF was significantly greater in men at 25% of VC, but similar between sexes throughout the remaining portion of the forced expiration. There was a significant effect of sex on ∆VGC, but not specific lung volume where differences were observed. The practical implication of these observations is that TGC artifact impacts variables used to assess pulmonary function to a similar extent between men and women, with the exception of FEF at 25% of VC, which was underestimated to a greater extent by the presence of more TGC in men.

### 4.1 Sex differences in TGC during forced expiration

The magnitude of TGC during forced expiration is primarily determined by three factors: (a) the density of the respired gas (Cross et al., 2018); (b) the magnitude of P_ALV_; and (c) the absolute volume of gas within the lung at the time of compression (Ingram & Schilder, 1966; Jaeger & Otis, 1964). In this study, we did not change gas density over the course of the expiration. As such, we expected that, because of greater expiratory muscle strength (Chen & Kuo, 1989) and larger static (absolute) lung volumes (Table 1), men would display a greater amount of TGC during forced expiration than women. Our findings support this hypothesis, insofar as P_ALV_ was systematically greater, and the difference in the sum total A_EX_ between MEFV_raw_ (TGC‐affected) and MEFV_corr_ (TGC‐corrected) curves larger, in men compared with women (Figures 4 and 6c). Importantly, to identify precisely *where* this larger TGC artifact occurred throughout forced expiration, we examined ∆FEF, ∆VGC, and ∆A_EX_ in 10% volume increments across the VC range. In so doing, we were able to determine that TGC artifact was larger in men during the first 25% of forced expiratory volume. However, ∆A_EX_ was greater in men at 65% and 75% of VC and ∆VGC was, on average, greater in men than in women irrespective of lung volume. Thereafter, TGC artifact was greatest early in the forced expiration and, in general, remained greater in men than in women throughout the expiration. Importantly, however, ∆FEF was only different at 25% of VC meaning that the effect of TGC on measured FEFs was comparable between sexes through the majority of the forced VC maneuver.

It is interesting that TGC artifact was not greater in men over a larger range of VC than what was observed given their greater expiratory P_ALV_’s (Figure 6c) during forced expiration. We did find multiple lung volumes where ∆A_EX_ was greater in men than in women, as well as a greater ∆VGC in men regardless of lung volume, but we expected more systematic sex differences across all three measures of TGC, particularly ∆FEF. Indeed, visual inspection of Figure 5 suggests that although ∆FEF, ∆VGC, and ∆A_EX_ appear—on average—greater in men than in women over the midcapacity range, the within‐group variability for these measures of TGC artifact was much larger in comparison. It is at least conceivable that, had we obtained direct measurements of TGC artifact via body plethysmography, and not derived its magnitude from the “maximal perimeter” MEFV curve, we may have observed larger effect sizes across these ranges of forced expired volumes. Thus, until more direct measures are obtained, we cannot rule out the possibility that TGC artifact was indeed larger in men compared with women across the midcapacity range. It would be of great interest for future investigations to examine sex differences in TGC artifact when data were plotted as a function of absolute lung volume, as opposed to a fraction of VC as we have done in this study.

### 4.2 Practical implications

It is well‐known that TGC artifact during forced expiration has important implications for the correct interpretation of pulmonary function. For example, if the MEFV curve is not adjusted for TGC, one is likely to: (a) underestimate pulmonary function at rest (Ingram & Schilder, 1966; Sharafkhaneh et al., 2004); (b) overestimate the degree of expiratory flow limitation during exercise (Guenette et al., 2010; Mota et al., 1999); (c) underestimate the efficacy of bronchodilator therapies (Sharafkhaneh et al., 2007); and (d) misconstrue the interpretation of pulmonary function when sojourning at altitude (Cross et al., 2018). Our data are consistent with Point 1 above, insofar as we observed significantly lower indices of pulmonary function when the MEFV_raw_ (TGC‐unadjusted) curve was used (Table 2). It is important to note, however, that while TGC artifact was present in all participants during forced expiration, its magnitude varied widely between individuals; for example, total ∆A_EX_ ranged between 0.4 and 9.4 L^2^·s^−1^ for all participants regardless of sex. As such, it is unlikely that a single, group‐level adjustment for TGC is adequate when attempting to account for its effects on dynamic pulmonary function, such as expiratory flow limitation during exercise(Duke et al., 2014; Tanner, Duke, & Stager, 2014; Weavil et al., 2015). In which case, any attempt to measure and, subsequently, adjust data for TGC‐artifact on a subject‐by‐subject basis is preferable.

Perhaps the most important finding of this study was that men experience a greater underestimation of FEFs only at 25% of VC compared with women despite the other measures of quantifying TGC demonstrating more sex differences. Therefore, studies examining the effect of sex on various parameters associated with flow–volume curves should keep in mind the disproportionate effect of TGC in men compared with women. For example, comparing the magnitude of expiratory flow limitation during exercise between men and women would be problematic if no effort is made to correct MEFV curves for TGC on an individual subject basis. In this situation, the magnitude of expiratory flow limitation reported in men may be inflated to a greater extent than women. A similar argument is forwarded for studies which calculate “ventilatory capacity” using the MEFV curve and tidal volume during exercise (Babb & Rodarte, 1993; Johnson, Scanlon, & Beck, 1995). Another potential consideration related to the lung volume‐dependence of TGC artifact is operating lung volumes during exercise. Visual inspection of Figure 5 illustrates that TGC in women was predominantly in the midcapacity range, while men displayed TGC (∆A_EX_ and ∆FEF) at the very beginning of forced expiration that did not wane until very close to residual volume.

### 4.3 Methodological considerations

It must be acknowledged that our measures of TGC were indirectly obtained by consideration of the “maximal perimeter” MEFV curve (i.e., MEFV_corr_) with respect to the *raw*, uncorrected MEFV envelope (i.e., MEFV_raw_). The ability of the “maximal perimeter” curve to quantify the magnitude of TGC relies heavily on the quality, number, and range of graded VC maneuvers performed, but, most importantly that a wide range of complete efforts are recorded. We encouraged subjects to vary their expiratory effort during successive VC maneuvers. Moreover, we were vigilant in reviewing flow–volume and pressure–volume data in real time so as to provide feedback to each participant, particularly when we identified that specific degrees of “effort” that were not well‐represented. Nevertheless, it is difficult for an individual to be able to perfectly deliver an expiratory effort at, for example, “60% of maximum” in a continuous manner from total lung capacity to residual volume. Therefore, it remains possible that our “maximal perimeter” MEFV envelopes were not *completely* free of TGC artifact. Given that our subjects performed, on average, 24 expiratory efforts (range = 10–40 efforts), we are confident that our “maximal perimeter” MEFV envelope captured the vast majority of TGC artifact present in our participants. To minimize the effect of incompletely measuring TGC artifact via this method, we processed our data by volume binning our measures of TGC (i.e., ∆FEF, ∆VGC, and ∆A_EX_) into 10% volume increments across the VC range. Notwithstanding this approach, it would be prudent to repeat these experiments while participants are seated inside a body plethysmograph, whereby TGC may be directly and continuously assessed throughout forced expiration (Ingram & Schilder, 1966). We did not account for menstrual cycle phase in our female subjects. Previous work has suggested that peak expiratory airflow differs across the menstrual cycle (da Silva et al., 2006) so this could have contributed to our findings.

## 5 CONCLUSIONS

We sought to quantify whether there was an effect of sex on the magnitude and location of TGC during forced expiration. We observed that TGC was present in all subjects, regardless of sex. However, we found that men displayed a larger magnitude of TGC compared with women throughout the forced expiration. With respect to the typical variables used to describe pulmonary function, it was only FEF at 25% of VC that was underestimated to a greater extent in men due to TGC. In general, failure to account for TGC results in a comparable underestimation of FEFs in men and women *after* the initial 25% of VC has been exhaled. Our findings also indicate that this greater magnitude of TGC artifact may be attributed to systematically larger expiratory P_ALV_’s produced throughout forced expiration in men compared to women. Our data do not allow us to comment on the role that differences in absolute lung volumes (presumably higher in men) played in mediating the larger TGC artifact in men, specifically during the first 25% of forced expiration. In general, these findings substantiate the claim that investigators must be cognizant of the impact that TGC bears on the interpretation of pulmonary function, bronchodilator efficacy, and magnitude of mechanical ventilatory constraints, particularly if the experimental design is to compare data between sexes.

## CONFLICT OF INTEREST

The authors have no conflicts of interest.

## AUTHOR CONTRIBUTIONS

EAG, TJC, BEC, and JWD designed the study. EAG, BEC, AWB, DSM, CLC, LEH, and JWD enrolled subjects and conducted data collection. EAG, TJC, CLC, LEH, and JWD analyzed the data. EAG, TJC, and JWD drafted the manuscript. All authors had complete access to all of the study data and critically revising the manuscript. All authors approved of the final version of the manuscript and take responsibility for the integrity of the data and accuracy of the data analysis.

## DATA REPOSITORY

Data can be found on Dryad at http://doi.org/10.5061/dryad.sxksn030c.
